# Variability of genome-wide DNA methylation and mRNA expression profiles in reproductive and endocrine disease related tissues

**DOI:** 10.1080/15592294.2017.1367475

**Published:** 2017-11-27

**Authors:** Nilufer Rahmioglu, Alexander W Drong, Helen Lockstone, Thomas Tapmeier, Karin Hellner, Merli Saare, Triin Laisk-Podar, Christine Dew, Emily Tough, George Nicholson, Maire Peters, Andrew P Morris, Cecilia M Lindgren, Christian M Becker, Krina T Zondervan

**Affiliations:** a Wellcome Centre for Human Genetics, University of Oxford, Roosevelt Drive, Oxford, OX3 7BN, UK; b Endometriosis CaRe Centre, Nuffield Department of Obstetrics & Gynaecology, John Radcliffe Hospital, University of Oxford, Oxford, OX3 7BN, UK; c Competence Centre on Health Technologies, Tartu, Estonia and Women's Clinic, Institute of Clinical Medicine, University of Tartu, Tartu, Estonia; d Department of Biostatistics, University of Liverpool, Liverpool, OX3 7BN, UK

**Keywords:** DNA methylation, endometrium, endocrine, endometriosis, gene expression, reproductive

## Abstract

Genome-wide association studies in the fields of reproductive medicine and endocrinology are yielding robust genetic variants associated with disease. Integrated genomic, transcriptomic, and epigenomic molecular profiling studies are common methodologies used to understand the biologic pathways perturbed by these variants. However, molecular profiling resources do not include the tissue most relevant to many female reproductive traits, the endometrium, while the parameters influencing variability of results from its molecular profiling are unclear. We investigated the sources of DNA methylation and RNA expression profile variability in endometrium (n = 135), endometriotic disease tissue (endometriosis), and subcutaneous abdominal fat samples from 24 women, quantifying between-individual, within-tissue (cellular heterogeneity), and technical variation. DNA samples (n = 96) were analyzed using Illumina HumanMethlylation450 BeadChip arrays; RNA samples (n = 39) were analyzed using H12-expression arrays. Variance-component analyses showed that, for the top 10–50% variable DNA methylation/RNA expression sites, between-individual variation far exceeded within-tissue and technical variation. Menstrual-phase accounted for most variability in methylation/expression patterns in endometrium (*P*_m_ = 7.8 × 10^−3^, *P*_e_ = 8.4 × 10^−5^) but not in fat and endometriotic tissue; age was significantly associated with DNA methylation profile of endometrium (*P*_m_ = 9 × 10^−5^) and endometriotic disease tissue (*P*_m_ = 2.4 × 10^−5^); and smoking was significantly associated with DNA methylation in adipose tissue (*P*_m_ = 1.8 × 10^−3^). Hierarchical cluster analysis showed significantly different methylation signatures between endometrium and endometriotic tissue enriched for *WNT* signaling, angiogenesis, cadherin signaling, and gonadotropin-releasing-hormone-receptor pathways. Differential DNA methylation/expression analyses suggested detection of a limited number of sites with large fold changes (FC > 4), but power calculations accounting for different sources of variability showed that for robust detection >500 tissue samples are required. These results enable appropriate study design for large-scale expression and methylation tissue-based profiling relevant to many reproductive and endocrine traits.

## Introduction

Genome-wide association studies (GWAS) are uncovering genetic variants involved in the pathogenesis of female reproductive disorders and traits.^1^^,^^2^ For instance, for endometriosis—a complex disease defined by the presence of endometriotic tissue outside the uterus associated with pelvic pain and sub-fertility—^3^ recent large-scale GWAS have identified 15 genetic loci robustly associated with the disease.^4–10^ Subsequent analyses demonstrating effects of endometriosis-associated variants on fat distribution (waist-to-hip ratio adjusted for body mass index, WHRadjBMI) indicate that many of these variants are likely also to impact the regulation of endocrine and metabolic processes.^11^^,^^12^ Typically, GWAS variants are located in inter-genic regions, and understanding their functional role requires molecular profiling (e.g., gene-expression assays) of tissues relevant to the conditions.^13^^,^^14^ Such integrated analysis of genomic, transcriptomic, and epigenomic data form the premise of large-scale tissue-based molecular profiling initiatives, such as GTEx,^15^^,^^16^ and the NIH Epigenome Roadmap.^17^ Endometrium is not included in these genomic profiling initiatives, an omission that prevents translation of genetic findings into results that could inform biomarker and drug target discovery programs for many common endometrium-related disorders, including endometriosis, infertility and recurrent miscarriage, abnormal bleeding, and endometrial cancer.

Endometrium is a heterogeneous and dynamic tissue composed mainly of glandular and luminal epithelial and stromal cells that are under strong cyclic endocrine influences,^18^ posing important questions regarding the possible variability of molecular profiling data generated. To date, 18 studies have investigated genome-wide mRNA expression in endometrium (11 in women with endometriosis), including samples from a range of 4 to 75 cases;^19–36^ only one recent study of 123 endometrium samples from healthy women assessed genome-wide expression quantitative trait loci (eQTL).^25^ There have been 5 genome-wide DNA methylation analyses (4 in women with endometriosis), including samples from 7 to 31 women.^37–41^ There is little overlap between differentially expressed transcripts/methylated genes identified in these studies, possibly due to: (1) variability in expression/DNA methylation patterns due to cellular heterogeneity inherent to whole-tissue analysis; (2) confounding variables unaccounted for that affect endometrial expression/DNA methylation profiles such as menstrual phase; (3) insufficient sample sizes that result in spurious or chance findings; (4) variation in tissue collection procedures and case/control definitions.

We aimed to investigate individual, tissue-derived and experimental variability of genome-wide DNA methylation and mRNA gene-expression profiles, and their implications for study design, contrasting 3 tissues: eutopic endometrium, endometriotic disease tissue (peritoneal disease, peritoneal lesions and ovarian disease, endometriomas) and subcutaneous abdominal fat. In addition, we determined sample sizes required to detect robust and biologically meaningful results from genome-wide whole-tissue expression and DNA methylation profiles for these 3 tissues, relevant to a broad range of reproductive, endocrine, and metabolic traits.

## Results

### DNA and RNA Quality

**Figure 1.**
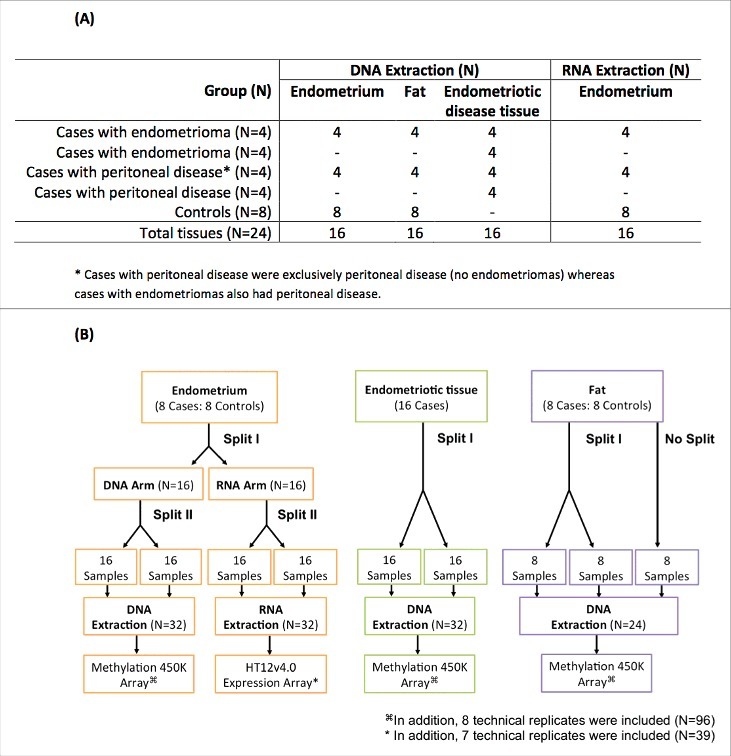
Experimental study design. Endometrium and subcutaneous abdominal fat from 8 cases and 8 controls, and endometriotic disease tissue from 16 cases (8 of which also contributed fat and endometrium) were used for DNA methylation analyses; the same endometrium tissue samples (n = 8) were also used for mRNA analyses. (A) Number of tissue samples from cases and controls processed for DNA and RNA extraction. (B) Tissue processing steps illustrating sample splits and technical replicates.

The experimental design included splits and replicates of endometrium, subcutaneous abdominal fat tissues collected from 8 endometriosis cases, and 8 controls and endometriotic disease tissue from 16 cases, which were used for DNA extraction; the same endometrium tissue samples were also used for RNA extraction (Fig. 1) (See Materials and Methods). All tissues provided good yields of DNA and RNA, with fat tissue providing the lowest yields [DNA mean = 533 ng, Standard Deviation (SD) = 344] and endometrium the highest (DNA mean = 4245 ng, SD = 1942; RNA mean = 4516 ng, SD = 3100) (Fig. S1). DNA quality was modestly associated with the initial yield (*P* = 0.03) and menstrual cycle day (*P* = 0.05), but not with tissue type (*P* = 0.38) or tissue weight (*P* = 0.99). A total of 94/96 (98%) of DNA samples passed quality control (>1000 ng DNA, >98% call rate), with probe intensities uniformly distributed for all 94 samples (Fig. S2).

RNA RIN-scores from endometrium did not correlate with initial tissue weight (*P* = 0.47) but did with RNA yield (*P* = 2.0 × 10^−3^), and cycle day on which the sample was collected (*P* = 1.2 × 10^−3^). Fourteen samples had RIN scores <7, indicating reduced quality; 10 of these (71%) were collected during the menstrual phase. These samples did not perform well in the microarray experiment (housekeeping gene signals <10,000, Fig. S3) and were excluded. The remaining 25 samples were of good quality (RIN > 7) with uniform distribution of probe intensities (Fig. S4).

### Variability in DNA methylation profiles

Variability in DNA methylation was investigated between-tissues using hierarchical cluster analysis and within-tissue variability was investigated using the DNA methylation profiles from each split and technical replicate of the tissues, through principal component analysis (PCA) for each tissue type separately (see Materials and Methods).

#### Between-tissue variability

**Figure 2.**
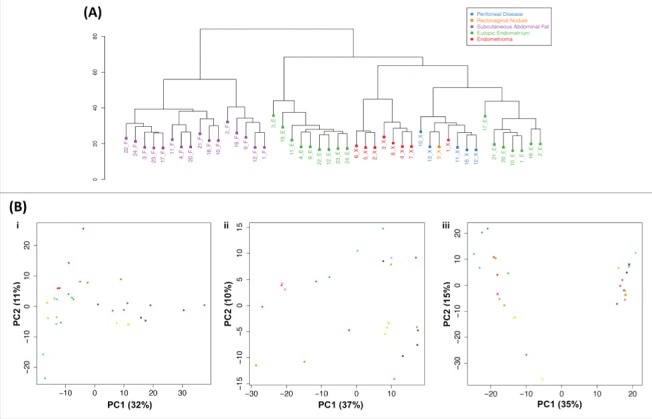
Variability in DNA methylation profiles from all 3 tissues. (A) Hierarchical clustering of DNA methylation profiles based on average methylation levels across splits and replicates for each sample (see Materials and Methods). Colors correspond to tissue type. (B) Principal component analysis (PCA) of genome-wide DNA methylation profiles derived from: i. endometrium; ii. subcutaneous abdominal fat; iii. endometriotic disease tissue. Each circle represents a sample; color coding designates different individuals. Same color circles designate sample splits; crosses (x) indicate technical replicates.

Genome-wide DNA methylation profiles for 94 DNA samples passing quality control (QC; see Materials and Methods) from endometrium and subcutaneous abdominal fat (cases and controls) and endometriotic disease tissue (cases) were analyzed. Clear clustering of DNA methylation profiles by tissue type was observed (Fig. 2A). Peritoneal disease tissue and endometrioma samples formed 2 distinct clusters.

#### Within-tissue variability

We investigated within-tissue (cellular heterogeneity) variability, using DNA methylation profiles of same-sample splits, and contrasted replicates of splits to detect contribution of technical (experimental) variation (see Materials and Methods). The PCA of DNA methylation profiles for each tissue showed close clustering of splits and replicates of the same sample, demonstrating that between-individual variation was greater than within-tissue (cellular heterogeneity variation and technical variation). Within endometriotic disease tissue samples, peritoneal disease and endometrioma samples again clustered separately—mainly on principal component 1 (PC1) explaining 35% of the DNA methylation variance (Fig. 2B.iii)—suggesting distinctive DNA methylation signatures (see below).

#### Effect of covariates

We investigated the effect of specific covariates on DNA methylation profiles averaged across splits and replicates by testing for their association with the first 2 principal components that capture most of the variation in DNA methylation profiles (Table S1): age (mean = 34.8 years, range = 25–47), menstrual phase (7 proliferative, 9 secretory, 6 menstrual phase),^33^ smoking (33.3% current smokers), BMI (mean = 25.4, range = 20.2–34), WHR (mean = 0.80, range = 0.72–0.89), and weight of the original tissue sample (mean = 0.39 g, range = 0.09–0.65). Menstrual phase was significantly associated with DNA methylation profiles in endometrium (*P* = 7.8 × 10^−3^), and in peritoneal lesions (*P* = 1.3 × 10^−6^) but not with endometriomas or fat (Table S1; Fig. S5). Age was also significantly associated with DNA methylation profiles in endometrium (*P* = 9 × 10^−5^), as well as in endometriotic disease tissue (*P* = 2.4 × 10^−5^). Smoking was significantly associated with DNA methylation in fat (*P* = 2 × 10^−3^). Notably, case/control status was only significantly associated with variability of global DNA methylation profiles in fat (*P* = 4 × 10^−3^).

#### Exploratory differential methylation analysis

We investigated if specific differentially DNA methylated sites of large effect were driving the tissue-based clustering for endometrium and endometriotic disease tissue observed in Fig. 2. We first compared DNA methylation profiles for all 14 endometriotic disease tissue and 16 endometrium samples from cases, adjusting for menstrual phase and age (see Materials and Methods), revealing 27,493 significantly differentially methylated probes, after Bonferroni multiple-testing correction, corresponding to 8,133 independent genes. The differentially methylated probes were concentrated within gene bodies (41%), inter-genic regions (31%), and regulatory regions (28%); in the context of CpG islands, they were mostly in open sea (58%) followed by shores (21%), CpG islands (10%), and shelves (11%) (Fig. S6). When we restricted the analysis to 8 paired eutopic and endometriotic disease tissue samples (linear model) from the same cases, after multiple-testing correction, 3,915 significantly differentially methylated probes in 1,860 independent genes were identified. Of the 3,915 significant probes, 3,858 were also significantly differentially methylated in the unpaired analysis. The top 30 differentially methylated probes are given in Table S4.

Pathway analysis using PANTHER^42^ on the 8,133 genes from the unpaired results showed significant enrichment in: (1) WNT signaling pathway (Bonferroni *P* adj = 7.49 × 10^−8^); (2) Angiogenesis (*P* adj = 4.12 × 10^−4^); (3) Alzheimer disease presenilin pathway (*P* adj = 4.79 × 10^−4^); (4) Cadherin signaling (*P* adj = 9.29 × 10^−4^); (5) Integrin signaling (*P* adj = 2.09 × 10^−3^); (6) Gonadotropin-releasing-hormone-receptor pathway (*P* adj = 3.65 × 10^−3^); (7) CCKR signaling (*P* adj = 6.22 × 10^−3^) (Table S5). PANTHER analysis on the paired results confirmed significant enrichment in WNT signaling (*P* adj = 2.14 × 10^−3^), Gonadotropin-releasing-hormone-receptor pathway (*P* adj = 1.75 × 10^−3^) and CCKR signaling (*P* adj = 4.82 × 10^−2^) (Table S6). We also tested the 27,493 differentially methylated probes for enrichment in histone modification ChIP peaks (H3K4me1, H3K4me3, and H3K27Ac, marks of open chromatin) and DNase I hypersensitivity sites in 127 different cell types in the Roadmap release 9 data sets.^17^ The probes were significantly enriched for H3K4me1 histone modification sites in 97 out of 127 cells types, but no other significant enrichment was observed for the other sites (Fig. S7).

Differential DNA methylation analysis of peritoneal lesions vs. endometriomas, adjusting for age (see Materials and Methods), revealed 31 differentially methylated probes significant after multiple-testing correction, 27 hypomethylated and 4 hypermethylated. Most (19/31) were located within gene bodies or regulatory regions of *MN1, SLC1A7, MYH7, DACT2, DHRS7, EPS8L1, ADHFE1, NFYB, FOXD2, CACNA1H, NCOR2, ZBTB7A, LAMB3, AGRN, UBE2CBP, BCAR1, CMIP* (Table S7).

Unsurprisingly, given the sample size, differential DNA methylation analysis of endometrium between cases and controls did not show any differentially methylated probes after multiple-testing correction.

#### Quantification of DNA methylation variability using variance component analysis

For each tissue type, the proportion of different sources of variances contributing to total phenotypic variance was estimated using variance component analysis (See Materials and Methods). When considering all CpG methylation probes in endometrium (Fig. S8a), irrespective of their variability, the distribution of technical variance (between replicates) was the main source of variation; similar results were observed for fat and endometriotic disease tissue (Figs. S9 and S10). However, these results included many probes with no or very low variability. When we considered the 50% and 10% most variable probes, a reverse pattern was observed, with between-individual variance explaining a high percentage of the overall variance, and a low within-tissue (split) variance, suggesting a low tissue-based heterogeneity of DNA methylation profiles (Fig. S8c). Mean variance estimates for the top 10% vs. 50% most variable probes were 0.84 (SD = 0.18) vs. 0.65 (SD = 0.29) for between-individual variance; 0.18 (SD = 0.14) vs. 0.23 (SD = 0.22) for within-tissue (tissue heterogeneity); and 0.06 (SD = 0.09) vs. 0.19 (SD = 0.23) for technical variation, respectively. Patterns for endometriotic disease tissue and fat were similar (Table S8).

### Variability in RNA expression profiles of endometrium

**Figure 3.**
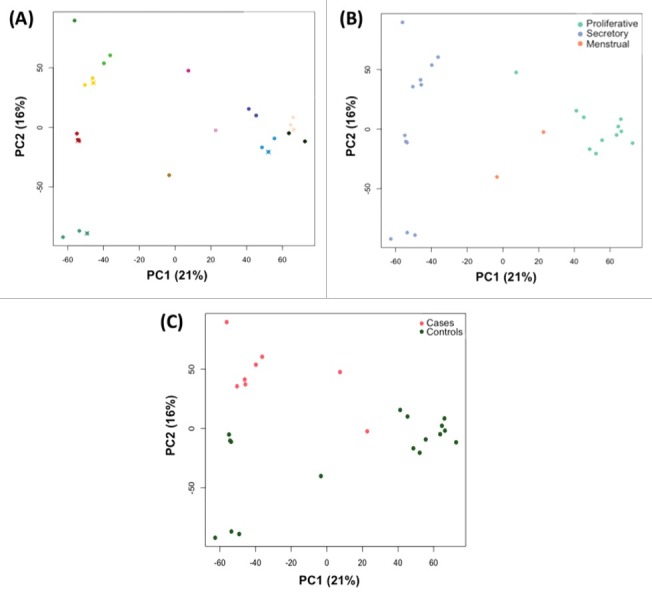
PCA analysis of RNA profiles in endometrium, showing total variation in profiles explained between PC1 and PC2. Each circle represents a sample; color coding designates (A) individual woman [same color circles represent the sample splits and crosses (x) indicate technical replicates]; (B) menstrual phase according to calculated cycle day based on self-reported LMP; (C) case/control status.

We similarly investigated tissue heterogeneity and technical variation in RNA expression profiles from 25 endometrium samples passing QC (12 individuals: 5 cases and 7 controls). Close clustering of splits and replicates of samples from the same individual was clearly observed (Fig. 3A), illustrating that between-individual variation exceeded within-tissue and technical variation.

### Effects of potential covariates

Fig. 3 shows clustering of samples by the first 2 principal components, PC1 (21.4% of variation) and PC2 (15.8% of variation), color coded by covariate status. Menstrual phase clustered mainly on PC1 *(P = 8.4* × 10^−5^) (Fig. 3B; Table S1), with samples from the secretory (cycle day 15+) and proliferative phases (cycle day 8–14) forming 2 distinct clusters. Clustering was also observed on the basis of case/control status on PC2 *(P = 7.4* × 10^−5^) (Fig. 3C; Table S1). No other covariates were associated with RNA expression profiles (Table S1).

### Differential expression analysis between endometriosis cases and controls

We performed differential expression analysis between cases and controls accounting for menstrual phase (see Materials and Methods), to identify the top contributing genes. After multiple-testing correction, 32 probes were significantly differentially expressed (Table S9), 28 upregulated and 4 downregulated. The fold-change (FC) in expression was >4 in 3 loci: *CYP26A1* (FC = 5.63, *P = 5.8* × 10^−6^, *P* adj = 0.025), *LRRC26* (FC = 4.49, *P = 6.6* × 10^−6^, *P* adj = 0.026), *LOC389816* (FC = 4.48, *P* = 5.6 × 10^−6^, *P* adj = 0.025).

### Quantification of RNA expression variability using variance component analysis

Similar to DNA methylation variance component analyses, when considering all genes probed by the RNA expression array irrespective of probe variability, the main source of variation was technical (Fig. S11a). However, when we considered the 50% vs. 10% most variable expression sites (Figs. S11b and S11c), the between-individual variance became the dominating component, although technical and within-tissue variation was larger than observed for DNA methylation patterns. Mean proportion of variance estimates for the top 10% vs. 50% most variable probes (Table S10) were 0.48 (SD = 0.39) vs. 0.40 (0.37) for between-individual variance, 0.27 (SD = 0.24) vs. 0.25 (SD = 0.25) for within-tissue variance, and 0.30 (SD = 0.25) vs. 0.40 (SD = 0.29) for technical variation, respectively.

### Power calculations

To determine the sample size needed to detect biologically relevant differential DNA methylation and expression between cases and controls, we conducted power calculations based on the 222,456 CpG-sites (80% power, α = 0.05/222,456 = ∼2 × 10^−7^) and 11,464 expression-probes (80% power, α = 0.05/11,464 = ∼4 × 10^−6^) for endometrium, where between-individual variance of DNA methylation/RNA expression exceeded within-tissue and technical variance (see Materials and Methods). **Fig. 5**A-C shows the results for power curves of 3 sample sizes for differential DNA methylation in endometrium and fat, and expression in endometrium between cases and controls, and **Fig. 5**D for detection of differential DNA methylation between paired endometriotic lesion tissue and endometrium from cases. For example, a 2% (β = 0.02) difference in DNA methylation with 80% power between cases and controls in endometrium can be detected with a sample size of 100 in 67,968/222,456 (30%) probes, with a sample size of 500 in 172,947/222,456 (78%) probes, and with a sample size of 1000 in 204,720/222,456 (92%) probes in endometrium (Fig. 5A). Most common effect sizes observed in DNA methylation studies of complex disease/traits range between 0.01–0.10 β value differences and gene expression ranges between 1–3-fold-change.^13^^,^^16^^,^^17^^,^^43^^,^^44^

## Discussion

This is the first study that systematically investigated the different sources of variability influencing DNA methylation and RNA expression profiles in tissues relevant to many reproductive, endocrine, as well as metabolic traits. The results have direct implications for studies including these profiles in terms of experimental design and sample size requirements.

We showed that in DNA methylation profiles from endometrium, endometriotic disease tissue, and subcutaneous abdominal fat, and in RNA expression profiles from endometrium, the between-individual variation is greater than within-tissue variation (tissue heterogeneity) and experimental (technical) variation when considering moderate to highly variable sites. This is reassuring for tissue-based profiling studies, given that most disease-association studies are focused on DNA methylation or RNA expression sites that at least show moderate variability across individuals in a population. Limiting to the top 10% most variable probes, the between-individual variation was 84.2% for endometrium, 84.2% for endometriotic disease tissue, and 79% for abdominal fat tissue. In expression profiles from endometrium, the between-individual variation (47.6%) also dominated over within-tissue (27%) and experimental (30%) variation in the top 10% most variable probes, although experimental variability was clearly more pronounced in expression profiling. This difference in contribution of experimental variability (6% vs. 29%) is probably expected because of the less stable nature of RNA compared with DNA, but it highlights the importance of using standardized protocols for sample collection and tissue processing, as well as adopting good experimental design in gene expression experiments.^45^

Overall, these results underline the application of whole-tissue profiling in these tissues to detect robust DNA methylation and expression differences between individuals, but they do not negate the importance of research focusing on profiling of distinct subcellular components, e.g., stromal and glandular epithelial cells in endometrium. Indeed, comparison of tissue-based with cell-specific profiles is crucial to understanding underlying regulatory biologic mechanisms. However, cell-type specific profiling is not without its own limitations (including potential for disturbance of expression profiles caused by the isolation protocols, and lack of scalability to larger studies). Our results suggest that for low-variability DNA methylation and RNA expression sites, cell-specific studies of sufficient size are particularly important, because tissue-based (cellular) heterogeneity and technical variability are likely to hinder the detection of biologically interesting variability.

Our results showed that menstrual phase is an important covariate and potential confounder in the analysis of differential DNA methylation and RNA expression in endometrium, as previously reported,^33^^,^^39^^,^^41^ and also in DNA methylation of peritoneal lesions—a novel finding. However, its effect on variability of DNA methylation in endometriomas and fat is much less pronounced. A recent study including 17 endometriosis cases (4 proliferative, 7 early secretory, 6 mid-secretory phases) and 16 controls (6 proliferative, 5 early secretory, 5 mid-secretory phases) suggested that DNA methylation changes across the menstrual cycle are associated with changes in gene expression for many loci associated with endometriosis, and that the greatest DNA methylation differences were observed in the mid-secretory phase.^38^ It is therefore important to investigate: (1) expression and DNA methylation profiles of endometrium in cases vs. controls; (2) DNA methylation profiles of peritoneal lesion vs. other tissues, adjusted or matched for menstrual cycle phase.

Age was a factor strongly influencing DNA methylation profiles in both endometrium and endometriotic disease tissue. There is abundant literature on DNA methylation differences associated with age, and the importance of age-related environmental influences.^46–49^ The age effect we observed could signify either population-based differences in exposures between women of different ages or the cumulative effects of lifetime exposures on DNA methylation sites. This highlights the need to ascertain in a larger study what these age-related differences constitute and how they might relate to endometrial biology and disease processes. Interestingly, smoking was an important factor influencing variability of DNA methylation in fat tissue—a novel finding. It is well established that smoking affects DNA methylation^50^ and many epigenetic markers related to smoking have been identified in blood including in inflammatory diseases, such as rheumatoid arthritis.^49^^,^^51–54^ Moreover, there is some evidence, which suggest smoking as a protective factor for endometriosis^55^ and endometrial cancer;^56^ however, the evidence is conflicting^57^ and requires further investigation. Intriguingly, endometriosis case/control status also influenced variability of DNA methylation profiles in fat tissue. We showed previously that there is significant sharing in the genetic etiology of endometriosis and fat distribution.^12^ Our findings suggest differential gene regulation in fat tissue between endometriosis cases and controls that warrants follow-up in a larger study.

We have shown that DNA methylation profiles of endometrium, endometriotic disease tissue, and abdominal fat are distinctly different, and that 2 different endometriosis tissue types—endometriomas and peritoneal lesions—have different DNA methylation signatures. Despite the relatively small sample size, differential DNA methylation analysis between endometrium and endometriotic disease tissue identified a set of differentially methylated genes, which were significantly enriched for 7 pathways, the top being *WNT* signaling—a pathway involved in sex-hormone homeostasis and reproductive tract development previously associated in genome-wide association analyses with endometriosis and in shared genetic regulation between endometriosis and fat distribution.^12^ Other pathways enriched for differential DNA methylation between endometrium and endometriotic disease tissue were angiogenesis, cadherin signaling (involved in cell adhesion and inflammation), and gonadotropin-releasing-hormone-receptor pathway. This suggests that genetic pathways perturbed by genetic variation in endometriosis are also dysregulated in terms of DNA methylation profiles. To what extent these DNA methylation differences are genetically driven or reflect differences in extra-uterine milieus, and how these might influence disease processes, needs to be explored in an adequately powered study. Differential DNA methylation analysis between endometriomas and peritoneal lesions identified 31 significantly differential methylated sites, though a limitation here is the potential for peritoneal cellular contamination driving some of these differences. All our differential DNA methylation analyses were exploratory and findings need to be replicated in larger studies.

We observed 32 differentially expressed RNA probes comparing endometrium between endometriosis cases and controls, after multiple testing correction. *CYP26A1* was upregulated >5-fold in cases compared with controls and is of particular interest since it is a progesterone-regulated gene ^58^^,^^59^ that has been found also to be upregulated in the proliferative phase for rAFS stage III/IV cases vs. stage I/II ^19^, while it was found to be nominally downregulated in endometriosis cases vs. controls in the secretory phase.^22^ The limited number of cases and controls within each cycle phase in this study prohibited us from performing between-phases or within-phase differential expression analyses. *CYP26A1* encodes for a retinoic acid metabolizing enzyme, and maps close to a region of the genome identified to be significantly linked to endometriosis in family-based analyses.^60^ Follow-up of the role of this locus in endometriosis is warranted in a larger study, in the context of menstrual phase.

A major limitation in deciphering the epigenomic and transcriptomic landscape of endometrium is that this tissue is not part of any large-scale genomic annotation projects such as ENCODE 61 and NIH Epigenome Roadmap.^17^ For example, histone modification sites and DNase I hypersensitivity sites provide information regarding enhancer regions in the genome. Enhancer regions play a central role in driving cell type-specific gene expression and enrichment of DNA methylation marks in these regions informs us about their potential functional role. We conducted our DNA methylation marks enrichment analysis in 127 different cell types (none relevant to endometrium). H3K4me1 histone modification was enriched across the majority of cell types, suggesting this as an enriched mark that is relatively non-specific to cell type. H3K4me1 histone modification is globally linked to distal regulatory regions.^62^ Genomic annotation data for endometrium and its constituting cell types is needed to allow a more informative exploration of these results.

Our power calculations show that tissue-based differential DNA methylation and expression studies require sample sizes of at least 500 tissues to detect the most common effect sizes expected for complex disease in these tissue types. For example, with sample size of 500 tissues, we can detect 2% DNA methylation difference between cases and controls in 172,947/222,456 (78%) CpG-sites in endometrium, 209,480/255,402 (82%) sites in fat, and between endometrium and endometriotic disease tissue in 393,584/483,422 (82%) sites. Most common effect sizes observed in large-scale complex traits DNA methylation studies range between 1–10%.^17^^,^^43^^,^^44^ This sample size also allows for detection of 1.5-fold change in gene expression of 9,500/11,464 (83%) expression-probes in endometrium. Large effect sizes could be detectable with sample sizes >100; however, this is unlikely to allow the discovery of most common mechanisms robustly. Reaching these samples sizes requires collaboration between centers that collect phenotypic data and biologic samples in a standardized manner. The WERF Endometriosis Phenome and Biobanking Harmonisation Project (EPHect) has provided standard operating protocols for collection, preprocessing and storage of tissues including endometrium, along with standardized questionnaires for phenotypic data collection,^45^^,^^63–65^ which allows such data to be generated and robust studies to be performed.

In summary, our study has identified parameters affecting DNA methylation and RNA expression variability in tissues relevant to many reproductive and endocrine research studies. It provides essential guidance for studies aimed at integrated analysis of genomic, transcriptomic, and epigenomic data in endometrium, which have become staple approaches for other tissues in different diseases and will aid further understanding of the biologic mechanisms underlying endometrium-related disorders.

## Materials and methods

### Recruitment, tissue, and data collection

Samples and data were derived from ENDOX, a prospective study focused on endometriosis and endometrium-related conditions, in which women undergoing a laparoscopy at the Endometriosis CaRe Center, John Radcliffe Hospital, Oxford (UK), are recruited. All data collection instruments and standard operating procedures (SOPs) used in ENDOX are in accordance with WERF EPHect standards^45^^,^^63–65^ (See Supplementary Note). Ethics approval for ENDOX was obtained from the NRES Committee South Central-Oxford Research Ethics Committee (09/H0604/58).

We selected 16 endometriosis patients and 8 healthy controls with regular menstrual cycles (predictable within 1 week), who had not used hormones at least 3 months before recruitment (Table S2). Controls were symptomatic [i.e., undergoing laparoscopy either for pelvic pain (n = 4), or subfertility (n = 4)] but no endometriosis was found during laparoscopy. Four controls were diagnosed with non-endometriotic adhesions, one with a non-endometriotic cyst, and 3 had no obvious pathology. Controls were frequency-matched to cases on menstrual phase (Table S3). Menstrual cycle was categorized based on self-reported last menstrual period (LMP) and cycle day at time of surgery, adjusted for cycle length,^33^ into menstrual phase (1–7 days), proliferative phase (8–14 days), secretory phase (15+ days); the menstrual phase was confirmed by RNA expression profiles (See Statistical analysis section).

### Experimental design

Figure. 1 shows the experimental design. A 100–120 mg section of endometrium and subcutaneous abdominal fat from 8 cases and 8 controls, and endometriotic disease tissue from 16 cases (8 of which also contributed fat and endometrium) were used for DNA methylation analyses; the same endometrium tissue samples (n = 8) were also used for mRNA analyses (Fig. 1A). To investigate within-tissue (cellular heterogeneity) variability of DNA methylation and RNA expression profiles, each of the 16 endometriotic disease tissue and endometrium samples and 8 of the 16 fat samples were split in random order before extraction (Fig. 1b). DNA and RNA extractions were conducted again in random order. To further allow for investigation of technical (array-related) variability, 8 replicates of the same DNA and 7 replicates of the same RNA sample were included on the arrays. Samples were run on arrays in random order.

### DNA extraction and methylation analysis

Genomic DNA was extracted from 88 samples (Fig. 1) using Qiagen DNeasy Kits and quantified by PicoGreen. Six samples with a yield >1 µg but concentrations lower than 16.5 ng/µl were concentrated by SpeedVac™ (Thermo Scientific). Whole-genome DNA methylation analysis was performed for 96 samples (including 8 replicates) using Illumina Infinium HumanMethylation450 BeadChip arrays, following the Illumina HumanMethylation450 protocol;^66^ and arrays were imaged using an Illumina HiScan™ scanner.

### RNA extraction and expression analysis

RNA was extracted from 32 endometrium samples (Fig. 1) using Qiagen RNeasy Kit and quantified using Nanodrop. RNA integrity (RIN) scores were obtained for each sample using Agilent Bioanalyser, before whole genome gene-expression analyses (39 samples, including 7 replicates) using Illumina Human HT12v4.0 Expression Bead arrays; arrays were imaged using an Illumina iScan Scanner.

### Statistical analyses

#### DNA and RNA Quality

DNA quality was measured as %CpG-sites detected at the 1% detection *P* value cut-off; RNA quality was assessed using RIN-scores. DNA/RNA quality was correlated with initial yield, menstrual cycle day, and tissue weight using Pearson's correlations, and with tissue type using one-way ANOVA linear models.

#### Principal component analysis (PCA) and hierarchical clustering

To investigate clustering of samples based on DNA methylation or expression profiles, PCA was performed for each tissue type separately, using the *prcomp* package with default settings in R (3.2.5). Hierarchical clustering based on DNA methylation profiles was performed using the *hclust* package in R. Similarly, the hierarchical cluster analysis (complete linkage based on Euclidean distance) identified 2 sample outliers (X_14, X_15), which are likely tissue sample swaps that were excluded from any downstream analyses. The univariate association between the PCs explaining most of the variance and different parameters (e.g., menstrual phase, age, smoking, BMI, WHR) was assessed using Pearson correlations and linear models. For 3 individuals with self-reported cycle days that were close to cycle categorization cut-offs (Table S1), cycle phase was re-classified via PC-based expression profile clustering.

#### Variance component analysis

For each tissue type, the proportion of different sources of variances contributing to total phenotypic variance was estimated for each probe using a linear mixed-effects model fitted by maximum likelihood. The estimated variances were extracted with the *VarCorr* function implemented in *lmer* in the R package *lme4*, and were decomposed into between-individual, within-tissue, and residual (technical) variance. More detailed methods are provided in Supplementary Note 1.

#### Differential DNA methylation analysis

Differential DNA methylation analysis was performed on the average of probe intensities across splits and replicates of 46 independent samples (16 endometrium; 16 fat; 14 endometriotic disease tissue) at each single CpG-site (483,422 total) by fitting linear models to all DNA methylation probes in R. We have conducted the following differential DNA methylation analyses using linear regression: (1) Endometrium from cases (n = 16) vs. all endometriotic disease tissues (n = 14) adjusting for age and menstrual phase (Unpaired analysis); (2) Endometrium from cases (n = 8) vs. all endometriotic disease tissues (n = 8) (Paired analysis); (3) Ovarian (endometriomas) (n = 8) vs. peritoneal disease tissues (n = 6) adjusting for age; (4) Endometrium from cases (n = 8) vs. controls (n = 8) adjusted for menstrual phase and age; (5) Fat from cases (n = 8) vs. controls (n = 8) adjusted for menstrual phase and age. *P* values were adjusted for multiple-testing using Bonferroni correction. The significantly differentially methylated genes were followed-up by PANTHER pathway analysis 42 and enrichment analysis in histone modification sites and DNaseI hypersensitivity sites from NIH Epigenome Roadmap R9 data set^17^ (See Supplementary note).

#### Differential expression analysis

Differential expression analysis was performed on the average of the probe intensities across splits and replicates of the 12 independent samples. We investigated differentially expressed genes in endometrium between cases and controls through linear model fitting adjusting for menstrual cycle phase, using *limma* in R.^67^ *P* values were adjusted for multiple-testing using Benjamini and Hochberg's method at the 5% false discovery rate (FDR) level.^68^

#### Power calculations

Power to detect differential expression or DNA methylation was assessed using *pwr.t.test* in R for 3 example sample sizes: n = 100; n = 500; n = 1000. The significance level (α = 0.05) was adjusted for the number of genes and DNA methylation sites found exhibiting differential expression/DNA methylation between individuals as defined percentage change in methylation mean (β value), and the fold change in expression mean (See Supplementary Note).

## Figures and Tables

**Figure 4.**
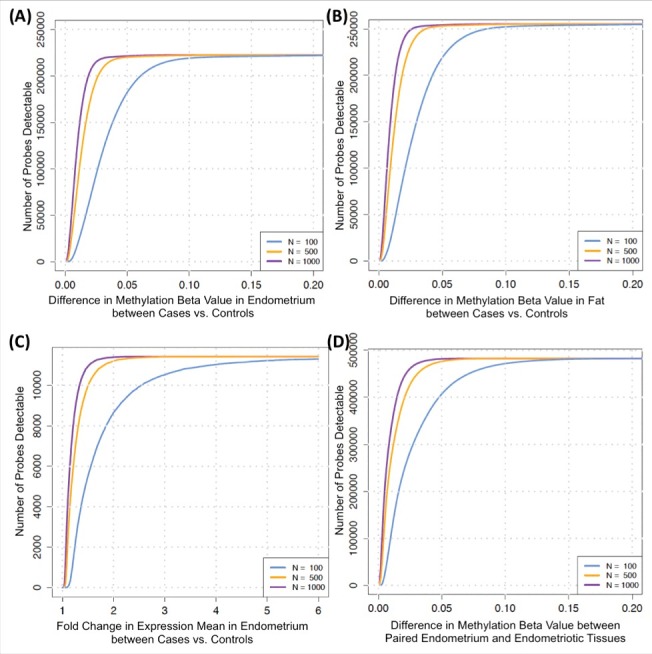
Power curves (80%) for 3 sample sizes (using the estimated between-individual variances) to determine the detectable (A) number of methylation probes with a difference in methylation values (β range 0–0.2) between cases and controls in endometrium; (B) number of methylation probes with a difference in methylation values (β range 0–0.2) between cases and controls in fat tissue; (C) number of expression probes with fold change in mean expression levels ranging between 1 and 6, between cases and controls in endometrium; (D) number of methylation probes with a difference in methylation values (β range 0–0.2) in cases, between endometriotic disease tissue and endometrium.

## Data Availability

The data sets used and/or analyzed during the current study are available from the corresponding author on request.

## Notes

*Footnote:* The power calculations to detect differential DNA methylation/RNA expression between cases and controls in endometrium and fat analysis are based on probes where between-individual variance of methylation/expression is more than within-tissue and technical variances (Fig. 4A-C). Whereas, Fig. 4D present power calculation results for detection of differential methylation between paired endometrium and endometriotic disease tissues coming from the same cases across all probes; hence, the scale of probes on y-axis includes all probes.
